# Regional and socioeconomic inequalities in access to pre-primary education in India: evidence from a recent household survey

**DOI:** 10.1186/s40723-023-00117-4

**Published:** 2023-04-24

**Authors:** Pradeep Kumar Choudhury, Radhika Joshi, Amit Kumar

**Affiliations:** 1 Zakir Husain Centre for Educational Studies, School of Social Sciences, Jawaharlal Nehru University, New Delhi, India; 2 Post-Doctoral Fellow, Indian Institute of Science, Bangalore, 560 012 India

**Keywords:** Pre-primary education, Regional inequality, Socioeconomic inequality, Household expenditure, India

## Abstract

In India, the National Education Policy 2020 recommends ensuring universal access to high-quality early childhood care and education for children aged 3–6 years by 2030. Using the 75th round of National Statistical Office data (2017–2018), this paper analyses the regional and socioeconomic inequalities in access to pre-primary education. Also, we investigate the specific role of households’ economic status and educational attainment in explaining these inequalities. We find considerable regional (rural/urban) and socioeconomic inequalities in access to pre-primary education in India, with girls and children belonging to historically disadvantaged social groups (scheduled castes and scheduled tribes) less likely to attend early childhood education, particularly in rural areas. We find that a substantial portion of the rural–urban gap in access to pre-primary education can be removed by controls for households’ economic condition and household head’s educational status. In addition, we find gender and socioeconomic inequalities in the household investment in early years education. These findings highlight the need to put policy efforts and commitments to reducing barriers to accessing pre-primary education for children in disadvantaged conditions in India.

## Introduction

A wealth of research, particularly in developed countries context, has demonstrated that investment in early years of education can have long-lasting benefits for children's cognitive development (Berlinski et al., 2009; Kaul et al., 2017; Kim et al., 2021; Rao et al., 2021). It also helps them in getting better employment opportunities in the labor market (Almond et al., 2018; Blanden et al., 2016; Flores et al., 2020; Heckman, 2011), and is considered an indispensable foundation for lifelong development and learning (Government of India, 2013; UNESCO, 2006; UNICEF, 2019). It is evident that children equipped with quality early childhood education are better prepared for the transition to primary school and set the stage for a positive transformation in learning outcomes throughout a child’s lifetime, and children from disadvantaged groups stand to benefit the most from this (Heckman et al., 2010; UNESCO, 2006; World Bank, 2018; Zhao et al., 2022). The research in several developing countries also shows the significant benefits of early childhood education (ECE) for the economy and society (Alcott et al., 2020; Zaw et al., 2021). For instance, Berlinski and Galiani (2007) study in the context of Argentina finds that the implicit childcare subsidy induced by the increased access to pre-primary schools increased maternal employment in the country. In Ethiopia, policy reforms to increase equitable access to pre-primary education (PPE) is positively associated with children’s reading skills in Grades 2 and 3 of primary school (Kim et al., 2021). Other studies in Bangladesh (Aboud et al., 2008), Indonesia (Hasan et al., 2013), Vietnam (Watanabe et al., 2005), Cambodia (Rao et al., 2012), Ecuador (Rosero & Oosterbeek, 2011) and Mozambique (Marinez, Naudeau & Pereira, 2012) also find that pre-primary school attendance positively affects exposure on cognitive skills, including language, numeracy and psychomotor development. Overall, we find considerable evidence on the importance of early years of human capital on societal wellbeing.

In India, Annual Status of Education Report (ASER) 2020 study finds a clear relationship between children's cognitive skills and their ability to do early language and early numeracy tasks (ASER Centre, 2020). Kaul et al. (2017) study in three Indian states (Assam, Rajasthan and Telangana), covering 14,000 children in the age group of 4 to 8 find that preschool participation in early years has a considerable impact on children’s school readiness and children who were exposed to activities for cognitive development in their preschooling scored better in the school readiness assessment. Given the importance of early childhood education on primary stage of education, the study recommends for including preschool as part of the Right to Education (RTE) Act (2009) that had made elementary education (class 1–8) a fundamental right for every child between the ages of 6 and 14 in India. Using Young Lives India longitudinal data, Singh and Mukherjee (2019) find that children entering preschool before the age of 4 have better cognitive achievement and subjective wellbeing when they reach the age of 12 in India. Under the age of 6 years, one child out of every five from around the world resides in India, which inventively plays a critical role in influencing the global status of children's development and learning (Kaul & Day, 2021).

The global education policy debates have increasingly focused on providing quality pre-primary education for all as a means to retain more students in primary education and improve learning outcomes (Bendini & Devercelli, 2022; Gove et al., 2018; UNICEF, 2019; Zhao et al., 2022). For instance, target 4.2 of the Sustainable Development Goal (SDG) aims to ensure universal “quality early childhood development, care and pre-primary education” to make children ready for primary education by 2030 (UN, 2015). UNESCO (2019) global report on “A World Ready to Learn: Prioritising quality early childhood education” states that pre-primary education must move from the margins of education sector plans to their centre to achieve universal pre-primary education. With the increasing recognition of the short-term and long-term effect of preschool education on children’s cognitive and non-cognitive abilities, several developing countries, including India, are focusing on ECE in their educational policies. For instance, the National Education Policy 2020 has emphasised the benefits of early years of education for children and recommends ensuring universal access to high-quality pre-primary education for children aged 3–6 years in India as soon as possible and no later than 2030. The aim is to ensure that all students entering Grade 1 are school ready in the country. The policy has specified that investment in early years of education enables children to participate and flourish in the educational system throughout their lives, particularly in the domains of physical and motor development, cognitive development, socio-emotional-ethical development and the development of communication and early language, and numeracy (MHRD, 2020). Also, the National Education Policy (NEP) 2020 acknowledges that quality ECE is not available to millions of young children in India, particularly for the socio-economically disadvantaged groups, and calls for strong investment in early childhood care and education (ECCE) initiatives to reduce inequality in access.

However, despite its importance, pre-primary schooling has largely been neglected by policymakers in India. The Right to Education Act (RTE) 2009, which was the most important act with respect to education in India, focused on universalising school for children aged between 6 and 14 years, but there was no consorted effort to increase enrolment for children between 3 and 6 years of age except suggesting that all children should have had some pre-primary education before enrolling in the primary grade. The analysis of 2020–2021 Unified District Information System for Education Plus (UDISE +) data on pre-primary education reveals several interesting facts: (a) only about 30% of the primary schools in India have pre-primary section, 25% of primary schools having Anganwadi Centre (AWC) in the school campus, and half of the primary schools are having either AWC or pre-primary sections in the school campus, (b) close to 60% of the private unaided recognised primary schools have pre-primary sections while this share is 20% in government primary schools, and of the total student enrolment in pre-primary education in India, more than two-third (67%) are in private schools, (c) less than one-third of the students admitted in grade 1 are having preschooling exposures from formal preschools while 18.3% have this exposure from AWCs, together adds up to 51%, (d) around 51% of the children admitted to grade 1 in private schools have exposure to preschools while this is only about 20% government school-going children (UDISE, 2021).

Zaw et al. (2021) find considerable gender, regional and socioeconomic inequality in pre-primary education access in developing world. Also, there is an inadequate understanding among both parents and service providers in India of how best to support young children’s cognitive development through early childhood education (Alcott et al., 2020). In this context, using the latest nationally representative household survey data on education, this paper provides an overview of pre-primary education in India, highlighting the grave disparities in access to preschools in the country. We have also examined the family investment in early years of education and how it varies with the socioeconomic settings of the households, particularly between rural and urban households. Kaul et al. (2017) study, in fact, suggests examining the regional and socioeconomic inequality in access to ECE using larger samples and diverse locations in India.

In the next sections of the paper, we will discuss (a) the policy development of pre-primary education in India, where an attempt is made to discuss the policy changes in the provisioning of pre-primary education in India and the major issues this sector is facing; (b) data and the econometric specifications; (c) results and discussion that covers determinants of access to early childhood education and household investment thereon; (d) policy implications and recommendations, and (e) conclusion which includes limitations of the study and the scope for future research.

### Pre-primary education in India: policy development and challenges

Pre-primary schooling in India is a combination of private schools and government-run centres known as Anganwadis. These Anganwadis are a network of community-based centres, run under the national flagship program of the Integrated Child Development Services (ICDS) scheme, sponsored by the Ministry of Woman and Child Development, Government of India. The scheme launched on 2nd October 1975 is one of the world’s largest and unique programmes for early childhood care and development, and currently there are about 1.3 million Anganwadi centres (AWCs) in India. It aims to provide preschool education to improve the nutrition and health status of children and also to reduce the incidence of malnutrition, morbidity, and mortality. Children between the age of 0–6 years, pregnant women and lactating mothers are the beneficiaries of this nationwide scheme.^1^ There are six main functions of Anganwadi workers (AWWs): supplementary nutrition, preschool non-formal education, immunisation, health check-up, nutrition and health education, and referral services. For instance, AWWs assist the primary health centre staff in implementing health component programmes like immunisation, health check-up of pregnant women, ante-natal and post-natal checks etc. They also assist in implementing nutrition programmes for adolescent girls.^2^

In addition, AWCs provide non-formal preschool education for children between 3 to 6 years of age. The ICDS is considered as the largest provider of ECCE services in India. In 2016, 38.7% of children, aged 3–6 years were attending the AWCs in India (Rao et al., 2021). Some Indian studies suggest that children who attend Anganwadis may on average have greater levels of cognitive development than those who do not (Arora et al., 2006; Samridhi et al., 2011). Alcott et al. (2020) find that parents consider Anganwadi as the first step in their childrens’ educational trajectories, and in a few cases, they treat this as a prerequisite for enrolment in primary school. Overall, access to AWCs is treated as a school readiness programme by many parents.

It is argued that although the Anganwadi network across India is huge, by and large, school readiness or early childhood development and education activities have not had high priority in the ICDS system (Banerji, 2019). AWWs are expected to participate in several non-teaching functions assigned to them (responsibilities such as vaccinations, maternal health and malnutrition), thus, do not get adequate instructional time for the young children enrolled in AWCs (Dhawan & Krishnan, 2019; Gupta et al., 2013). Maity (2016) finds that in West Bengal and Chhattisgarh (two eastern Indian states), the supplementary nutrition programme of ICDS is found to occupy most of the AWW’s time, it leaves significantly less time available to dedicate to preschool education. Also, the reduction in the duration of job and refresher training for AWWs has resulted in their reduced learning opportunities about preschool education. Evidence shows that children in Anganwadis do worse than private preschool children on cognitive and early language tasks like picture description (for instance, 14% of children in Anganwadis could recognise letters or more than 52.9% in private preschools) (Wadhwa, 2019). While children receive some pre-primary education even if inadequate by attending AWCs, the focus of the ICDS is more on establishing elements of wellbeing that are essential for children to learn, rather than focusing explicitly on providing opportunities to learn (Alcott et al., 2020). In fact, an important issue discussed in the NEP 2020 is the lack of training of Anganwadi workers/teachers in the curriculum and pedagogy of ECCE, and calls for imparting training for them in the areas of early literacy, numeracy, and other relevant aspects of ECCE. Overall, studies accessing the effectiveness of ICDS schemes have found that it does not perform well enough due to poor implementation strategies and lack of utilisation of resources, specifically in the provisioning early childhood education and getting children ready for school (Mohapatra et al., 2021; Wadhwa, 2019).

What is the policy attention towards the growth and development of early childhood education in India? Recognising the critical role of Early Childhood Care and Education (ECCE) on the holistic development of children, the National Policy on Education (1986), perhaps the most significant education policy in India so far, emphasised the provisioning of ECCE for children below 6 years. The suggestion was to integrate ECCE programme of the country with the ICDS. The 2009 Right to Education Act suggests state governments provide free preschool education for all children until they complete the age of 6 years as this would help them prepare for elementary education. The Ministry of Women and Child Development, Government of India approved the ‘National Policy on Early Childhood Care and Education’ in 2013. The target in this policy was to achieve holistic and active learning capacity of all children below 6 years of age by promoting free, universal, inclusive and equitable pre-primary education programme. In fact, a national curriculum for ECE was released soon afterwards based on the recommendations of this policy. However, its implementation on the ground has been sluggish (Alcott et al., 2020), even though the policy suggests periodic appraisal (each 5 years) to assess the progress of implementation. The NEP 2020 aims to universalise preschool education by 2030 that can ensure foundational literacy and numeracy for children below 6 years, and make them school ready. As a starting point, the policy suggests to strengthen existing Anganwadi Centres with high-quality infrastructure, play equipment, and well-trained Anganwadi workers/teachers. It also suggests smooth integration of early childhood care and education (specifically, Anganwadis) into school education in the long run. Overall, education policies of the last five decades in India, including the recent National Education Policy 2020, have emphasised the public provisioning of ECE. However, implementation of these policy targets on the ground has been slow and derisory.

The state's inadequate attention for the robust development of pre-primary education of children led to significant growth of the private sector in pre-primary education in India. There are about 300,000 private preschools across the country (Ministry of Women and Child Development, 2017). The recent UDISE + data (2020–2021) shows that of the total children attending pre-primary education in India, two-third are in private unaided recognised primary schools (UDISE, 2021). Also, the poor functioning of AWCs under ICDS has pushed vast majority of parents to send their children to private preschools (Dhawan & Krishnan, 2019; Majumdar et al., 2021). But, the expansion of private schools that mostly caters to the need for pre-primary education in India and enrol students from age 3 (even below this age in some cases) widens the socioeconomic inequality in access to early education. While children from disadvantaged communities largely take admission in Anganwadi Centres, children from socially and economically better-off families access privately managed pre-primary schools. This calls upon expanding and improving comprehensive early childhood care and education, particularly for children from socio-economically disadvantaged backgrounds.

In the Indian context, a few studies have looked at access to pre-primary schooling. For instance, Pal (2020) finds that boys in urban areas who do not belong to Scheduled Castes (SCs), Scheduled Tribes (STs) or Other Backward Classes (OBC)^3^ are most likely to attend preschool. Using data from the Young Lives Survey on enrolment in public versus private preschools in India, Singh and Mukherjee’s (2017) study found that more boys (51.2%) attend private preschools than girls (38.7%). Further, enrolment in private preschools was around 74% for upper castes, 46.6% for OBCs and 29% for SCs. Ghosh and Dey (2020) found that parents’ socioeconomic status plays a critical role in the choice of type of preschool in India. For instance, economically better off and educationally more aspirant parents prefer private preschools over public ones. In this context, Majumdar et al. (2021) argued that there is a need for public provisioning of early childhood education to achieve equity goals.

Until very recently, none of the surveys of the National Statistical Office (NSO) collected data on pre-primary education at the national level, thus limiting any kind of research on pre-primary education. The 75th round of National Sample Survey is the first round to have collected data on pre-primary school-going children in India. Using this data set, collected between July 2017 and June 2018, this paper tries to examine the regional and socioeconomic disparities in attendance in preschools in the Indian context. We believe that this is one of the first attempts to understand disparities in preschool education in the Indian context. Results from a logit estimation show us that compared to urban households, students in rural India have significantly less chance of accessing early years of education, and interestingly, this gap minimises with the increase in households’ capacity to pay and household head’s education status. Also, we find a clear inequality in the household investment in pre-primary education between rural and urban India.

The study has the following hypotheses: (a) students belonging to low socioeconomic strata are less likely to attend pre-primary education in India after controlling for other factors; (b) there exists a stark inequality in access to early years of education between rural and urban areas, and this gap varies significantly with households’ capacity to pay and household head’s education status.

### Data and econometric specification

#### Data

The study is a quantitative study and uses existing data collected by National Statistical Office (NSO) between July 2017–June 2018. We use the 75th education round titled "*Household Social Consumption: Education."* It is a nationally representative survey including a sample of 1,13,757 households comprising of 64,519 rural households and 49,238 urban households and enumerating more than 500,000 individuals. The focus of this round was to collect information on participation in education, family expenditure on education and the extent of educational wastage in terms of dropout and discontinuation. Importantly, the 75th education round is the first round to provide information pertaining to pre-primary schooling in India. In the survey, the question asked is: ‘*level of current enrolment in the basic course*’ for the household members of age 3–35 years who are currently attending education. One of the responses to this question is—a particular household member is currently enrolled in pre-primary (nursery/kindergarten,^4^ etc.). As the question in the household survey specifically asks about children’s enrolment status in nursery and kindergarten in each household, children accessing Anganwadi Centres (AWCs) under ICDS are not part of this survey as these centres do not offer formal pre-primary education courses like nursery and kindergarten. Therefore, one limitation of the data used for this study is that the children between the age of 3–5 years who are enrolled in AWCs are recorded as ‘not enrolled’ in the pre-primary education (nursery/kindergarten). Rao et al. (2021) have pointed out that there is data discrepancy on access to pre-primary education in India as a few data sources count the children attending AWCs while a few others exclude them. Thus, getting accurate data on access to pre-primary education in India is a challenge. The 75th education round data of NSO also provides information on socioeconomic and institutional settings of students currently attending pre-primary education and household spending on it. The analysis of regional and socioeconomic inequalities in access to pre-primary education in India in this paper is based on the information collected on the enrolment status of children in pre-primary education.

We have restricted the data to individuals surveyed in the age group of 3–5 years, the official age group of accessing pre-primary schooling in India, giving us a sample of 19,962 children. According to Government of India guidelines, these students should attend pre-primary education in 2017–2018, and would transit to the primary section (grade I) after age 5. However, we find that of the total sample of 19,962 children, 20.2% were attending pre-primary education (nursery/kindergarten) at the time of the survey in 2017–2018, 11.9% were attending primary schools, and 67.9% were neither attending nursery/Kindergarten nor in primary grade, and we put them in the category of ‘out of formal school’ (see Table 1). However, it is important to note that some children included in the ‘out of formal school’ category might have attended AWCs in 2017–2018. AWCs in 2017–2018. But the data used for this study (75th education round data of NSO) does not collect this information, which is an important data limitation. In India, children’s participation trajectories in the early years of education do not reflect the age or grade norms specified by the national educational policies, for instance, around 20% of children in Rajasthan are already attending primary school at age four (Alcott et al., 2020). The 2020–2021 UDISE + data (Ministry of Education, Government of India) show that 6% of the total enrolment in primary level of education (grade 1–5) are in the age group of less than 6 years old (UDISE, 2021).

**Table 1:** Share of children aged 3–5 years attending pre-primary, primary or no schools

Age	Pre-primary grade (nursery/kindergarten)	Primary grade	Out of formal school	Total
3 year	8.3	0.1	91.6	100 (6667)
4 year	25.5	1.4	73.1	100 (6904)
5 year	26.7	36.2	37.1	100 (6391)
Total (3–5 years)	20.2	11.9	67.9	100 (19,962)

Figures in parentheses in the last column show the absolute number of students

Source: Authors’ estimation from 75th NSO round data, 2017–2018

To analyse the regional and socioeconomic inequality in access to pre-primary education in India, we removed 2376 children who were attending primary schools (grade I and above) from the total sample of 19,962 children. Thus, the final sample for our analysis includes 17,587 children aged 3–5 years, who were either attending pre-primary education or not in 2017–2018.

#### Econometric specification

The main focus of this paper is to examine the factors determining access to pre-primary education in India. We have used a logit regression model where the dependent variable is the probability of attending pre-primary education among 3 to 5 age group children.^5^ It is a dummy variable that takes value '1' for the children currently attending pre-primary education and '0' if they are not attending pre-primary education, and the total sample includes 17,587 children in the age group of 3–5 years.

The econometric specification that we used in our study is as follows:

$$Y= \alpha +\beta \left(\mathrm{Head\,Education}\right)+\gamma \left(\mathrm{Income}\right)+\delta \left(\mathrm{Location}\right)+\theta {\varvec{X}}+\varepsilon ,$$

where, $\alpha$ is the intercept, while $\beta , \gamma ,and \delta$ are the coefficients of the main explanatory variables, ***θ*** is the coefficient vector of the other control variables, and $\varepsilon$ is the error term.*** X*** is the vector of the explanatory variables.

The explanatory variables used in the logit model are gender, caste, religion, region, household consumption expenditure—a proxy to annual family income,^6^ household head’s education status, and family size. The main variables of interest along which we examine the heterogeneity in the predicted probabilities of attending pre-primary education are location of the household, i.e., rural or urban, economic status of the family, and educational attainment of the heads of the households. Thus, we incorporate interaction of location with household head’s education status and household consumption expenditure (used as a proxy for the economic status). The justifications for including the explanatory variables in the regression model (backed up by the evidence in the existing literature) is discussed in the results section, where the interpretations of the respective logit coefficients are discussed. Summary statistics of the variables used in logit models are given in Table 2.

**Table 2:** Summary statistics of the variables used in the logit models

	All-India			Rural-India			Urban-India		
	NOB	Mean	SD	NOB	Mean	SD	NOB	Mean	SD
Ppeattend									
No	17,189	0.771	0.420	11,459	0.823	0.381	5730	0.603	0.489
Yes	17,189	0.229	0.420	11,459	0.177	0.381	5730	0.397	0.489
Gender									
Female	17,187	0.471	0.499	11,458	0.476	0.499	5729	0.454	0.498
Male	17,187	0.529	0.499	11,458	0.524	0.499	5729	0.546	0.498
Caste									
ST	17,189	0.104	0.305	11,459	0.124	0.330	5730	0.039	0.194
SC	17,189	0.222	0.416	11,459	0.243	0.429	5730	0.157	0.364
OBC	17,189	0.458	0.498	11,459	0.454	0.498	5730	0.470	0.499
General	17,189	0.216	0.412	11,459	0.180	0.384	5730	0.334	0.472
Religion									
Hindu	17,188	0.780	0.414	11,459	0.802	0.399	5729	0.710	0.454
Muslim	17,188	0.178	0.383	11,459	0.159	0.366	5729	0.241	0.428
Others	17,188	0.042	0.200	11,459	0.039	0.194	5729	0.049	0.216
Lnhhconsexp	17,189	11.552	0.554	11,459	11.436	0.503	5730	11.929	0.542
Headedn	17,189	6.292	4.335	11,459	5.685	4.059	5730	8.260	4.611
Familysize	17,185	5.997	2.556	11,455	6.105	2.597	5730	5.647	2.382
Location									
Rural	17,189	0.764	0.425	–	–	–	–	–	–
Urban	17,189	0.236	0.425	–	–	–	–	–	–

Source: Authors’ estimation from 75th NSO round data, 2017–2018

## Results and discussion

**Fig. 1:**
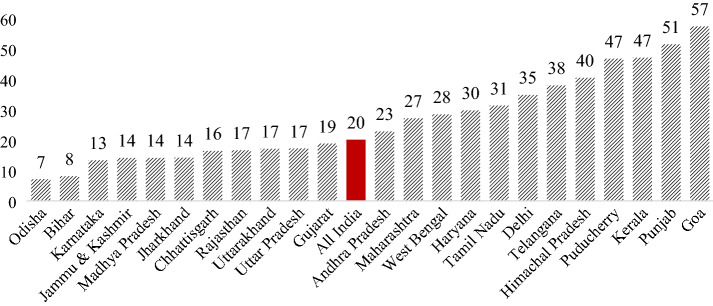
Percentage of children (3–5 years) attending pre-primary education by major states. Source: Authors’ estimation from 75th NSO round data, 2017–2018

Access to early years of education is quite limited in India. Estimates from the NSO data reveal that only around 20% of children in the age group of 3–5 years are attending pre-primary education in India in 2017–2018, leaving a majority of children out of formal school. Additionally, the share of children attending pre-primary education in India varies widely across different states (Fig. 1). In the states like Goa, Punjab, Sikkim and Tripura, more than half of the 3–5 years of children attend pre-primary education, while this share is less than 10% in backward states such as Odisha and Bihar. Therefore, at the macro-level, a direct relationship exists between economic conditions and participation in pre-primary education in India, and more discussion on this is done in latter part of the paper using household-level data from NSO. Nevertheless, to unravel the state-specific dynamics in the growth of pre-primary education, there is a need to examine how their respective social and policy contexts shape this sector. However, this analysis is beyond the scope of this paper as the objective of this paper is to provide an all-India picture of the regional and socioeconomic inequality in access to education.

The share of children attending early years of education also varies by their socioeconomic profile. A slightly higher percentage of male children (21.2%) are attending pre-primary schooling as compared to female counterparts (19%) (see Table 3). According to 2020–2021 U-DISE data, total enrolment in pre-primary level of education is 10.6 million, of which 5.7 million are boys and 4.9 million are girls (U-DISE, 2021). Being from a lower caste limits access to pre-primary education, as only 13.1% of scheduled tribes (STs) and 17.3% of scheduled caste (SCs) children attend pre-primary education compared to the corresponding figure of 29.4% for upper castes. Such inequalities tend to be higher amid rural areas than in urban areas. Similarly, differences exist in student enrolments by different religious groups such as Hinduism, Islam, Christianity, Sikhism, Jainism etc. Evidently, Muslim children in the age group of 3 to 5 are least represented in pre-primary education (16.9% attendance) compared to 20% in case of Hindus and 35.9% among other religions. The marginalisation and exclusion of Dalits and Muslims in the sociocultural, political, and economic spheres of India is often linked with their lower access to education which starts from very early years of schooling, as evident in our analysis. Similar to our results, Kim et al. (2021) find that girls (particularly in more disadvantaged regions) are less likely to attend pre-primary education while children from more advantaged backgrounds (those whose parents are literate, have reading materials at home, and live in urban areas) are more likely to participate in pre-primary schools in Ethiopia (p. 103).

**Table 3:** Percentage of population attending pre-primary education in India by age and socioeconomic characteristics

	3 years	4 years	5 years	Total (3–5 years)
Gender				
Female	8.5	24.1	24.8	19.0
Male	8.0	26.8	28.3	21.2
Caste				
ST	4.8	18.0	16.5	13.1
SC	7.4	20.8	24.1	17.3
OBC	7.0	24.2	24.5	18.7
General	13.2	36.8	38.7	29.4
Religion				
Hindu	8.4	25.6	26.0	20.0
Muslim	6.5	20.0	24.6	16.9
Others	12.9	47.2	48.3	35.9
Residence				
Rural	6.0	20.5	20.6	15.7
Urban	15.6	43.1	43.8	34.3
Consumption quintile				
Q1 (poorest)	3.2	13.1	16.0	10.8
Q2	7.2	18.2	21.1	15.5
Q3	7.5	27.9	26.4	20.4
Q4	11.5	36.5	35.3	27.8
Q5 (richest)	19.5	54.4	49.8	41.5
Household head’s education status				
Illiterate	4.3	16.2	16.8	12.4
Elementary	8.5	24.2	26.3	19.6
Secondary	9.4	34.8	38.0	27.5
Grad and above	21.9	48.6	43.9	38.1
Total	8.3	25.5	26.7	20.1

Authors’ estimation from 75th NSO round data, 2017–2018

We find a significant difference in children’s attendance in pre-primary education between rural and urban regions. Around 35% of the population in the age group of 3–5 years attend nursery and kindergarten in urban areas, while this is 15.7% in rural India. Likewise, access to early years of education varies widely with households' economic status, which is measured in terms of their monthly consumption expenditure. The share of population (in the age group of 3–5 years) attending pre-primary education increases with each successive consumption quintile. It ranges from 10.8% for the bottom quintile (Q1) to 41.5% for top quintile (Q5)—indicating a rich-poor gap of 30.7 percentage points. Besides, household head’s education status also matters in this context. The share of population attending pre-primary education increases with the increase in household head's education ranging from 12.4% (among illiterates) to 38.1% among those who completed graduate-level education or above.

### Socioeconomic determinants of pre-primary attendance: logit estimates

We estimate four logit regression equations. Equation 1 considers the simple analysis of the probability of attending pre-primary education by incorporating all the explanatory variables in the model, and the corresponding results are shown in Table 4. Equations 2 and 3 include additional interaction terms in the model, i.e., the interaction effect of location with household head’s education status (Eq. 2) and household consumption expenditure (Eq. 3). The rationale behind adding interaction terms in the model is to analyse how the probability of attending early years of education varies for the children of different socioeconomic setups in rural and urban India. However, the estimated odds ratios of the explanatory variables in the logit model help only in identifying the direction of the relationship with the outcome variable. Therefore, after estimating the odds ratio, this study has also calculated the marginal effects (margins) of predicted probabilities for analysing the interaction term effect.

**Table 4:** Predicted probability of attending pre-primary education (3–5 years): logit estimates

	Simple model		Models with interaction term	
	Equation 1		Equation 2	Equation 3
	Odds ratio	AME	Odds ratio	Odds ratio
Male	1.105*** (0.040)	0.018*** (0.006)	1.106*** (0.040)	1.101*** (0.040)
Caste (reference—STs)				
SC	1.162** (0.082)	0.025** (0.012)	1.147** (0.082)	1.178** (0.083)
OBC	1.250*** (0.083)	0.038*** (0.011)	1.236*** (0.082)	1.273*** (0.084)
Upper caste	1.544*** (0.109)	0.078*** (0.012)	1.540*** (0.109)	1.601*** (0.114)
Religion (reference—Hindu)				
Muslim	0.629*** (0.033)	− 0.077*** (0.008)	0.616*** (0.032)	0.628*** (0.033)
Other religions	1.515*** (0.103)	0.082*** (0.014)	1.488*** (0.102)	1.541*** (0.105)
Lnhhconsexp	1.935*** (0.086)	0.118*** (0.008)	1.966*** (0.088)	1.000*** (0.000)
Headedn	1.051*** (0.005)	0.009*** (0.001)	1.067*** (0.006)	1.053*** (0.005)
Familysize	0.922*** (0.008)	− 0.015*** (0.002)	0.920*** (0.008)	0.927*** (0.009)
Urban	1.556*** (0.066)	0.079*** (0.008)	2.026*** (0.154)	2.173*** (0.163)
Interaction effect				
Urban # headedn	–	–	0.965*** (0.008)	–
Urban # lnhhconsexp	–	–	–	1.000*** (0.000)
Constant	0.000*** (0.000)	–	0.000*** (0.000)	0.140*** (0.012)
Prob. > Chi^2^	0.000	–	0.000	0.000
Pseudo-R^2^	0.075	–	0.076	0.073
Observations	17,587	17,587	17,587	17,587

Standard errors in parentheses; ***p < 0.01, **p < 0.05, *p < 0.1; *AME* average marginal effect

Source: Authors’ estimation from 75th NSO round data, 2017–2018

Though several studies have examined the inequality in access to education (at different levels of education) by socioeconomic groups, specific discussion on pre-primary education is sparse. Therefore, it is important to examine what are the major socioeconomic barriers in accessing pre-primary schooling in India, and more importantly, how the effect of these factors varies between rural and urban India. Results reveal that gender plays a crucial role in household decisions to send their children for pre-primary education. Chances of attending pre-primary education are higher for boys as compared to girls. Being female decreases the chances of accessing pre-primary education by 1.8% than their male counterparts (see Table 4). Education for All (EFA) Global Monitoring Report 2007 (UNESCO, 2006) finds that in many underdeveloped and developing countries, girl children residing in rural areas or those in poorer households have significantly lower participation rates in ECCE programmes than their counterparts who are male, live in urban areas or belong to richer households. Gender inequality in education is a serious issue in India as parents often prefer to give quality education to their sons than daughters (Narwana, 2019; Sahoo, 2017). Studies also find pro-male gender bias in intra-household allocation of resources for education, particularly among poor households (Azam & Kingdon, 2013). In a patriarchal society such as India, girls are considered unmitigated potential expenditure, someone who is unlikely to contribute to the household income and whose marriage will take away a substantial part of her parent’s fortune as dowry (Sen & Seth, 1995). In addition, females in India face many disadvantages in the labour market, reducing the returns to investment in daughters’ education (Maitra et al., 2016).

Secondly, caste is a significant social phenomenon in India, and several studies have focused their attention on inequalities in education between social groups—caste and religion (Biswas et al., 2010; Basant & Sen, 2014; Sundaram, 2009; Tilak and Choudhury, 2019). It is often asserted that caste is an important determining factor in access to quality education in India (Jones, 2017; Narwana, 2019). The four caste categories in India include Upper Caste (UC), Scheduled Caste (SC), Scheduled Tribe (ST), and Other Backward Class (OBC). Though the Indian government has been making collective efforts since independence to bridge the socioeconomic gap between the advantaged and disadvantaged groups, SCs and STs have remained socially, economically and educationally deprived because of their specific occupational and geographical condition (Chauhan, 2008; Jodhka, 2016). Similarly, despite being a significant religious community, Muslims remain behind other Socio-Religious-Cultural (SRC) Groups in terms of education (Government of India, 2006; Hasan, 2012; Tilak, 2015). Several studies argue that educational backwardness has put Muslims in social, economic and political disadvantages (Alam & Kumar, 2019; Hasan, 2016). However, evidence on inequalities in pre-primary education participation across social groups (caste and religion) is meagre in India. Our results show a clear hierarchy among the people, with the predicted probability of attending pre-primary education in terms of the social groups. Compared to ST households, SCs, OBCs and forward caste households are 2.5%, 3.8% and 7.8% more likely to attend pre-primary education, respectively. This is apparent, as most SC/ST students come from lower or middle-class families and are unable to afford pre-primary education, particularly in privately managed preschools. Likewise, compared to Hindus, Muslims are 7.7% less likely to send their child for pre-primary education, whereas a child from other religions is 8.2% more likely to attend pre-primary education. According to Census (2011), the respective population shares are 80.5% (Hindu), 13.4% (Muslims) and 6.1% for other religions, including Christians, Sikhs, Buddhists, Jains.

Access to education differs considerably between rural and urban areas, arising due to the natural clustering of education institutions in and around metropolitan and urban areas. Students from rural areas do not have many options to choose from (which affects their education participation), whereas people from urban areas have moderate access to a variety of educational institutions, and hence, they seem to be able to access education according to their choice. While we do not have many studies that reveal the rural–urban inequalities in pre-primary education, Borooah (2017) reports that rural–urban gaps in access to education in India are high and have not diminished much in the last two decades. Compare to urban counterparts, students from rural areas were likely to experience greater difficulty in physically accessing further education in India (p. 29, ibid). In the present study, households' location (rural/urban) is also statistically significant in determining the probability of attending pre-primary education in India. The value of marginal effect associated with variable 'urban' reveals that individuals residing in urban areas have 7.9% higher chances of attending pre-primary education as compared to those from rural areas. This corroborates with the available literature concluding the dominance of urban areas in accessing education at different levels (Hasan & Mehta, 2006). Likewise, Zaw et al. (2021), using survey data from 83 developing countries between 2010 and 2016 found significant rural–urban gap in access to pre-primary education—the gap in access to pre-primary education between rural children (67.88%) and their urban peers (80.15%) is around 12.27 percentage points (p. 6).

Furthermore, it is not only the availability of opportunity that matters to participate in pre-primary education, several socioeconomic factors of the households are also important. Several studies in developing country contexts have established that educational inequalities between the rich and the poor are highly striking, and they have widened over the years (Borooah, 2017; Chakrabarti, 2009; Tilak, 2015; Tilak et al. 2019). Further, studies show that children of the rich and middle-income families in India are mostly attending private schools, and government schools are now largely accessed by the children of poor and lower-middle-income groups (Harma, 2011; Kumar & Choudhury, 2020; Tabarrok, 2013; Woodhead et al., 2013). Similarly, the demand for private schools is greater among higher educated parents than their less educated counterparts (Kumar and Choudhury, 2020; Tilak & Sudarshan, 2001). And this is mainly due to the increasing demand for quality education as several studies find that students enrolled in private schools learn better than their counterparts in public schools (Singh, 2015; Kumar and Choudhury, 2021). It is also found that educated parents in India spend more on their children’s education (Saha, 2013; Tilak, 2002). To understand how education and economic status of the household’s matter in accessing pre-primary education in India, the empirical results on the interaction of location with household head’s education status and household consumption expenditure (a proxy for economic status of the households) are discussed in detail in the following section.

### Effect of household head’s education and capacity to pay on access to pre-primary education: a disaggregate effect on rural and urban India

**Fig. 2:**
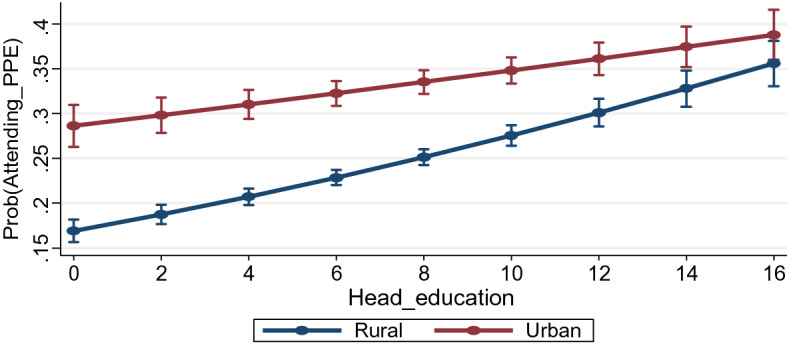
Predicted probabilities of attending pre-primary education by location and household head’s education status

**Fig. 3:**
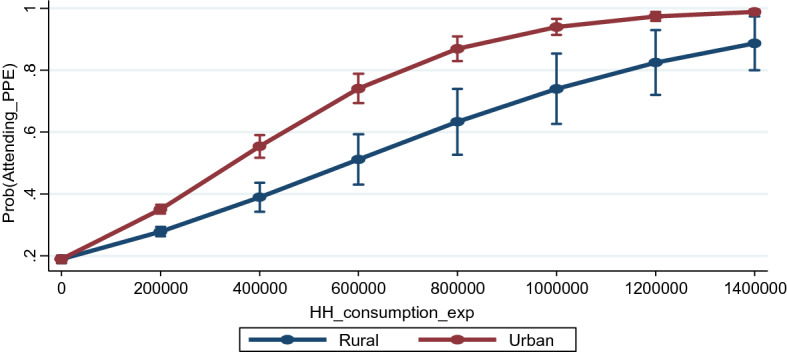
Predicted probabilities of attending pre-primary education by location and household consumption expenditure

This section analyses the results related to the sectoral gap in access to pre-primary education in India. How does the sectoral inequity in access to early years of schooling vary between poor and rich households? Does household head’s education status matter differently in rural and urban areas while taking decision to send their children to nursery and kindergarten classes? We analyse the changing effect of household head’s education status and household consumption expenditure on access to pre-primary by putting an additional interaction term in Eqs. 2 and Eq. 3, respectively. The results for all the equations presented in Table 4 show that both the interaction terms are significant in the model. Both head's education status and household consumption expenditure are positively associated with the likeliness of their child attending pre-primary education. Post estimation predicted probabilities have been calculated for both interaction effects after controlling for all other confounding factors in the model, and the results are presented in Figs. 2 and 3.

Table 4 shows that household head’s education status is positively related to attendance in pre-primary education in India. With a unit increase in the level of education of head, the probability of their child attending nursery and kindergarten classes increases by around 1%. How does the likelihood of accessing pre-primary education among the educated heads vary between rural and urban India? Fig. 2 presents the predicted probabilities of attending pre-primary education for rural and urban children at different levels of households’ head education. This has helped us to analyse the changing effect of head's education on sectoral differences in pre-primary education participation. It shows that the overall likelihood of attending nursery and kindergarten classes increases with an increase in head's education in both rural and urban areas. However, the marginal effect of increasing household assets is higher for rural children than their urban counterparts—the curve for rural students has a steeper slope than that for urban students. Moreover, the gap between the two probability curves narrows with an increase in head's education, and interestingly, these curves almost coincide with the highest level of head's education. Perhaps, the households with higher educational accomplishments tend to be more apprehensive (vis-à-vis the illiterates) about the future of their wards and hence allocate more resources towards the education of their children.

A survey of literature points out that although a few studies have mentioned that economic status of the household is a major barrier to access to different levels of education, academic interest to examine it at the pre-primary level has been relatively limited. In this study, households’ monthly per capita consumption expenditure is used as a proxy for their economic status, as NSO does not collect data on household or individual income. Estimates reveal that economic status of a household has a significant impact on the decision to send a child for accessing pre-primary education. The chances of attending early years of education increase with the increase in their paying capacity. Estimates show that a unit increase in household consumption expenditure increases the probability of attending pre-primary education by 11.8 percentage points. While we find a few studies that have established a positive and statistically significant association between a households’ economic status and the access to childrens’ education in India (Borooah, 2017; Chakrabarti, 2009; Tilak, 2015; Tilak et al. 2019), the evidence on how this magnitude differs across rural and urban areas is limited, specifically for pre-primary education. Here, we calculate predicted probabilities of attending pre-primary education for rural and urban households at different levels of household consumption expenditure (see Fig. 3). It shows that while the probability of sending a child for pre-primary education increases with household consumption expenditure in both rural and urban areas, the magnitude of increase in this likeliness is observed more in rural areas. The sectoral gap in attending pre-primary education increases with consumption expenditure but gradually declines among the rich households. Interestingly, the highest rural–urban gap is found among middle-class households. This indicates that resource constraints and social norms interact to manifest regional inequity in pre-primary education participation. When there are no budget constraints, then rural parents are equally likely to educate their children as urban households. However, a detailed comparative study between the poor and rich families would help in better understanding the regional dynamics of access to early years of education in India.

#### Household spending on pre-primary education in India

We find clear evidence that attendance in pre-primary schools varies widely with households’ capacity to pay. Here we discuss the inequality in household expenditure on early years of education by socioeconomic groups. It is expected that the quality of pre-primary education attended by students with different family backgrounds varies substantially, and the difference in family investment in it would help us to explain this phenomenon. Also, with an increasing presence of private sector in the provisioning of early years of education in India, it is important to look at the variations in the household expenditure on pre-primary education, in addition to examining the inequality in accessing it. Though there are few available studies on the inequality in household expenditure on different levels of education in India (Azam & Kingdon, 2013; Chandrasekhar et al., 2019; Duraisamy & Duraisamy, 2016; Tilak et al. 2019), to the best of our knowledge, there are no studies for pre-primary education. Thus, this analysis makes an initial foray into the relatively underexplored research agenda at the socioeconomic inequality in household investment in pre-primary education.

The annual average household spending on pre-primary education in India is reported to be around ₹9053 (about 122 US dollars^7^), accounting for 6.2% of the total annual household consumption expenditure in 2017–2018 (see Table 5). However, the spending varies by different socioeconomic and institutional factors. There is a pro-male bias in spending on early years of education—households spent relatively more on sons ($129) than daughters ($114)—per capita family expenditure on PPE as a share to total annual household consumption expenditure is 6.5% for male and 5.8 for female children. The extent of gender bias in spending is observed to be more in urban areas. This corroborates with the findings of many studies conducted on household spending at different levels of education in India (Azam & Kingdon, 2013; Chaudhuri & Roy, 2006; Duraisamy & Duraisamy, 2016; Kingdon, 2005; Saha, 2013;). Variations in household spending on the children currently attending nursery and kindergarten are also prevalent across social groups. Households from ‘forward caste’ incurred the highest expenditure ($173), followed by OBCs ($111), and as expected, SCs and STs spent the lowest, i.e., $92 and $58. This reports that forward caste households spent around 298% more than STs (gap of $115) and 188% more than SCs (gap of $81). Annual average family expenditure as a share to total household consumption expenditure is three per cent for ST while it is 8.8% for forward/general caste—a difference of 5.8%. Further, a significant rural–urban disparity exists in household spending on pre-primary education in India. Urban households spent 256% more on the early years of education of their children than rural counterparts. This inter-regional gap in spending was found to be slightly more in the case of males (261%) than females (248%). Rural households spend around 4% of their annual consumption expenditure per child on accessing pre-primary education, while this is 10% for urban households—2.5 times higher.

**Table 5:** Household spending ($) on pre-primary education in India by gender and location

	Gender		Location			Share to total HH consumption exp
	Female	Male	Rural	Urban	All India	
Gender						
Female	–	–	73	182	114	5.8
Male	–	–	80	208	129	6.5
Caste						
ST	49	67	38	130	58	3.0
SC	92	92	63	156	92	4.7
OBC	111	111	78	167	111	5.6
General	151	191	100	256	173	8.8
Residence						
Rural	73	80	–	–	77	3.9
Urban	182	208	–	–	196	10.0
Quintile						
Q1 (poorest)	41	42	38	69	42	2.1
Q2	60	65	60	74	63	3.2
Q3	80	101	85	108	92	4.7
Q4	137	135	120	152	135	6.9
Q5 (richest)	265	310	193	311	290	14.7
head education						
Illiterate	77	70	65	98	73	3.7
Elementary	79	96	68	136	88	4.5
Secondary	150	160	99	215	156	7.9
Grad and above	241	272	118	328	260	13.2
Medium						
Others	45	50	37	83	48	2.4
English	203	215	156	255	210	10.7
School type						
Government	15	14	11	29	14	0.7
Private	171	176	128	224	174	8.8
Total	114	129	77	196	122	6.2

The average spending on pre-primary education is higher for each successive expenditure quintile in 2017–2018. It is the lowest for the poorest households ($42) and highest for the richest households ($290). The top quintile households (quintile 5) spend close to seven times more on pre-primary education as compared to the bottom quintile (quintile 1). Poorest households (Q1) spend 2.1% of their annual household consumption expenditure (used as a proxy to measure economic status of the family) per annum per child for PPE, while it is 14.7% for the richest households (Q5). A similar expenditure pattern is observed across gender and location. The gap in spending between poorest (Q1) and richest (Q5) households is more among male (7.4 times) and in rural area (5.07 times) than their female (6.5 times) and rural counterparts (4.5 times). Likewise, households with better-educated heads tend to invest more in their children's early years of education than households with less-educated heads. For instance, households where the heads have completed graduation and above level spent $260 on pre-primary education of their children—remarkably higher (close to four times more) than households where heads are illiterate ($73). Annual average family spending on PPE as a share to total household consumption expenditure is only about 3.7% for households in which heads are illiterate, while this figure is as high as 13.2% for households where heads are highly educated (graduates and above). This clearly indicates a positive impact of household head’s education status on family investment in early years of education of the children. Apart from socioeconomic factors, another major factor that affects the household spending on early years of education is the type of school that the child is enrolled in. Households choosing private schools for their children’s pre-primary education spent 12.3 times more than those choosing government schools ($174 against $14). While the spending on pre-primary education as a share to total household consumption expenditure is less than one per cent for the children who attend government schools, it is 8.8% for private pre-primary school-going children. Similarly, households sending their children to English-medium pre-primary schools spend 437% more than those who opted for other medium preschools. These figures reflect the growing presence of the private sector in the provisioning of early childhood education in India and the escalating costs of accessing it.

The pandemic, which began in March 2020, has worsened attendance in pre-primary schools as only a small fraction of students can continue with pre-primary schooling online. There is also postponement of school admission of young children during the pandemic that has led to less enrolment of students in pre-primary education. In 2020–2021, enrolment of students in pre-primary level has reduced by 29.1 lakh and 18.8 lakh as compared to 2019–20 (UDISE, 2021). A recent study by Vidhi Centre for Legal Policy finds that access to ECE has reduced significantly during the pandemic, and interestingly, the attendance in ECE in virtual platforms has reduced by up to 60 per cent, compared to pre-pandemic situation (Vernekar et al., 2021). Inequality in access to ECE during school closure is alarming as the children from socially and economically disadvantaged households have almost lost touch with the early childhood care centres as majority of the students from these groups do not have access to devices and internet to continue their classes in virtual mode (ibid: 24). As children are expected to learn while at home during the pandemic, it is expected that the educational inequality in the early years of education will increase. Parents from low socioeconomic strata are often less-educated and hence find it difficult to engage with their children’s learning process. Further, in some cases, many parents from low-income households lost their jobs due to the pandemic, which has left many households incapable of investing in ECE. Thus, it is important to examine the magnitude of both short-term and long-term damage of COVID-19 on ECE sector in India. Examining this concern is even more important as NEP 2020 targets ensuring universal access to pre-primary education for 3–6 age group children by 2030.

Our results show that the percentage of children attending pre-primary education in India varies widely across different states. Majority of the economically better-off states (with per-capita Net State Domestic Product higher than the national average) have higher pre-primary attendance rates than their counterparts, i.e., states with low per-capita NSDP. Other factors that influence attendance in pre-primary school include gender (males are more likely to attend ECE) and being from a higher caste—only 13.1% of scheduled tribes (STs) and 17.3% of scheduled caste (SCs) population attend pre-primary education compared to the corresponding figure of 29.4% for upper-castes. Differences also exist in student enrolments by different religious groups and in particular Muslim children are least represented in pre-primary education in India.

Our findings also reveal a significant difference in children’s attendance in pre-primary education between rural and urban regions. Around 35% of the population in the age group of 3–5 years attend nursery and kindergarten in urban areas, while this is 15.7% in rural India. This gap may be due to a set of factors like lack of availability of preschools in rural areas, lack of information among parents and socioeconomic backwardness. Finally, the share of population attending pre-primary education increases with the increase in household head's education. In particular, a unit increase in the level of education of head, the probability of their child attending nursery and kindergarten classes increases by around 1%.

### Policy implications and recommendations

As India is getting the focus back on ECE with the target of achieving universal and equitable ECE (as envisioned in the NEP 2020), our study has considerable policy relevance. Findings from our study suggest that to achieve universalisation of preschool education, NEP 2020 must first build preschools that focus on providing education and are independent of all other community-enhancing projects that the Anganwadis presently cater to. Currently, AWWs organise non-formal preschool activities for children in the age group of 3–6 years, specifically helping them in designing and making toys and play equipment. However, AWWs are engaged in immunisation, health check-up, carrying out quick family surveys for the government, maintaining the birth and death records, organising social awareness programmes/ campaigns etc. It is now more critical than ever to improve the efficiency in which the ECE programmes are delivered in India. And an important channel to achieve this is to increase public investment in the provisioning of preschools in India. As majority of the children from socially and economically marginalised sections do not access ECE in India, there should be incentives for these households to send their kids to preschool.

Further, inadequate public investment in ECE has led to, to some extent, the mushrooming of budget-private preschools to cater the demand for low-income households, specifically in rural areas. This has resulted in increased inequality in access to quality preschools in India. We recommend for the state-led expansion of preschools in India as private preschools are costly, hindering the children from lower socioeconomic positions in accessing it. Given the wide regional and socioeconomic inequality in access to ECE in India, any further expansion of pre-primary education in the private sector would escalate the gap. In addition, through effective policy measures, there is a need to reach the target population and make them understand the positive impacts of pre-primary education on the future wellbeing of their kids. For instance, our findings show that less educated households are less likely to send their children to pre-primary education as compared to more educated households; thus, efforts should be made by the state to minimise this inequality. And, in the short-term, this can be done by creating awareness among less educated parents on the benefits of ECE for their children and in the long-term through larger public investment in education. Given the prolonged closure of schools and ECE centres in India amid pandemic, post covid- policy should focus on bringing back children to the ECE centres and minimise their learning gap. Selection of locations for setting public preschools is also crucial. Given the wide discrepancies in preschool attendance based on religion and caste, more schools must be built in areas where enrolment is presently very low. Finally, enforcing free and compulsory laws that make preschool compulsory would also go a long way in ensuring better learning outcomes for school-going children in India. The suggestion is to include pre-primary education as an integral part of the Right to Education Act (2009), which is currently for children aged 6 to 14.

## Conclusion

The focus of this paper is to examine the disparities in access to preschool education in the Indian context. We used a nationally representative household survey data to analyse the regional and socioeconomic inequality in access to pre-primary education in India. This study is perhaps the first one to analyse the socioeconomic and regional contours in access to ECE among Indian households in an empirical framework. In the context of an increasing presence of the private sector in the provisioning of ECE in India, we also offer an understanding of the costs of pre-primary education and how it varies for households of different socioeconomic steps accessing ECE. We find that household income, caste, location, household head’s education status and gender, all play an important role in influencing parents’ decision to send their children to preschool. Our analysis reveals widespread disparities in preschool education in India, particularly across rural and urban areas and across different castes. Not surprisingly, expenditure on preschool education is higher amongst households with higher income, and parents choose to spend more on preschool education of their sons than for daughters. A grave cause for concern is that our sample reveals that only 20 per cent of children within the age group of 3–5 years attend preschools. Furthermore, of the total enrolled students at pre-primary level of education, most children (71 per cent) attend private preschools, and a little more than half of the students are enrolled in English-medium pre-primary schools (NSO, 2020). These numbers suggest that preschools in India are still largely unavailable for most parents, especially in rural areas and with low-income levels.

However, findings of this study need to be interpreted in the light of certain limitations. First, the data used for this study (75th education round data of NSO) considers nursery/kindergarten as pre-primary education, and it does not include the children who attended AWCs in the survey year, i.e. 2017–2018. Our results show that around 67.9% were not attending nursery/Kindergarten, and we put them in the category of ‘out of formal school’. However, it is important to note that some children included in the ‘out of formal school’ category might have attended AWCs in 2017–2018. Including the children who were attending AWCs in the analysis would have given a different picture on access to preschools in India. Due to data limitations, we were unable to make a clear demarcation between children enrolled in *Anganwadi* centres and those enrolled in pre- primary schools and children who are not enrolled in either of these, which is a major limitation of the study. Second, access to variables for the empirical analysis is restricted as we have used a secondary data set. For instance, the study has discussed the demand-side factors in explaining the access to ECE in India, and we are largely unable to explore the supply-side interventions (e.g., availability of ECE centres, teachers, physical infrastructure etc.) as the data set used in the study does not collect those. Third, we analyse the inequality in access to ECE at national level in this study. But, understanding the issue at the regional level is critical as we see considerable variations in the provisioning of ECE in different states in India. Finally, as the data used for this study was collected in 2017–2018, we could not analyse the impact of ongoing COVID-19 pandemic on ECE sector in India, an important issue to explore.

In this study, we made an initial foray into understanding the socioeconomic contours in accessing pre-primary education in India. The study motivates future research examining the socioeconomic inequality in access to pre-primary education at the sub-national level. Future research could also address the equity implications of this inequality, as early gaps in learning and skills trap the children in lower developmental trajectories from which it becomes increasingly difficult to escape (World Bank, 2018). There is growing evidence that access to better quality pre-primary education can potentially reduce learning inequalities. But we know little about the effect of early years of education on the later learning outcomes, particularly in the context of India. This is an important area for future research. Future studies should also study the impact of COVID-19 pandemic on early childhood education in India. Given the prolonged closures of schools and ECE centres, it is critical to find out the unequal impact of the pandemic on learning and development of the children from different socioeconomic setups. More importantly, future research on comparing the learning and development of the children who access formal pre-primary schools (nursery/kindergarten) and AWCs could bear broad implications for inequality in access to early childhood education in India.

## Data Availability

This study is based on a national survey conducted by National Statistical Office (NSO) from July 2017 to June 2018, Ministry of Statistics and Programme Implementation, Government of India. The data is publicly available at: https://www.mospi.gov.in/web/mospi/download-tables-data/-/reports/view/templateTwo/16203?q=TBDCAT.

## Footnotes

1 Available at: https://icds-wcd.nic.in/icds.aspx (accessed 29th January 2021).

2 Role and responsibilities of AWWs can be found at: https://wcd.nic.in/sites/default/files/Roles%20and%20Responsibilities%20of%20AWWs.pdf (accessed 01 November 2021).

3 The caste system in India is an old social stratification primarily based on the rituals followed by different groups and their engagements with different occupations. Over the time, the discourse of caste has been shifted from ritual hierarchy and social discrimination to an instrument to mobilize people for economic and political gain. Currently, for administrative or official purpose the caste system in India is classified into four major groups namely Scheduled Castes (SCs), Scheduled Tribes (STs), Other Backward Classes (OBCs), and Upper Caste or General. It is well recognized that the SC, ST, and OBC households fare relatively poorly in several socio-economic indicators when compared with the general category households. Therefore, SCs, STs, and OBCs are given special treatments by the government of India in many welfare fields like education, health, and employment, etc. (Choudhury, 2016, p. 545).

4 Besides Anganwadi Centres (AWCs), children in the age group of 3 to 6 in India attend formal preschools (mostly in the private sector) for their pre-primary education, and this is widely referred as Nursery/Kindergarten (KG). Unlike AWCs, the main focus of Nursery/Kindergarten classes is to prepare children for formal primary education. In some school settings, the pre-primary education is for two years (Nursery and KG) while in a few schools it is for three years—Nursery, Lower kindergarten (LKG) and Upper Kindergarten (UKG).

5 Since a few children were in pre-school at age 6,7 and even 8 we tried running our models using a sample of children aged 3–8. Our results remain quite similar. However, since the appropriate age group for pre-primary schooling is 3–5 years, that is the sample that we retain for our analysis.

6 In this study, annual consumption expenditure of the household is used as proxy for their economic status as the NSO surveys do not collect data on family income.

7 The Indian Rupee (INR) is converted into US dollars (with an exchange rate: 1 USD = 74.21, the prevaling exchange rate data from the Reserve Bank of India at the time of data analysis) for easy understanding of the international readers.
